# Conventional MRI's diagnostic role in HTLV-1-associated myelopathy reframed

**DOI:** 10.1055/s-0045-1811520

**Published:** 2025-08-15

**Authors:** Abelardo Q. C. Araújo

**Affiliations:** 1 Universidade Federal do Rio de Janeiro, Instituto de Neurologia Deolindo Couto, Rio de Janeiro RJ, Brazil; 2 Fundação Oswaldo Cruz, Instituto Nacional de Infectologia Evandro Chagas, Rio de Janeiro RJ, Brazil; 3 Universidade Federal Fluminense, Programa de Pós-graduação em Neurologia e Neurociências, Rio de Janeiro RJ, Brazil

The diagnosis and management of HTLV-1-associated myelopathy/tropical spastic paraparesis (HAM/TSP) remains challenging in endemic countries. The classic spastic paraparesis syndrome develops in 5% of HTLV-1-infected patients. Still, the urologic symptoms occur in 40% of individuals who show no motor deficits. Thus, they are considered to have “probable HAM/TSP.” The study by Carvalho et al. provides a timely evaluation of spinal cord (SC) metrics on conventional T2-weighted MRI as a diagnostic tool in cases of definite and probable HAM/TSP. The main finding of the study —that basic cross-sectional metrics can detect lumbar spinal cord atrophy in suspected HAM/TSP patients —holds significant implications for clinical practice and health systems with limited access to more modern imaging technology. HAM/TSP diagnosis through MRI has historically relied on two main subjective findings: thoracic SC atrophy and T2 hyperintensities. 1 2 These findings are unreliable and often absent, particularly during the initial stages of HAM/TSP and its oligosymptomatic phase. The present study introduces three crucial findings that represent its original contribution to the field: the measurement of SC area through manual segmentation at 1.5-Tesla MRI proves more sensitive than subjective visual assessments for detecting changes; the atrophy pattern in HAM/TSP patients extends from the thoracic to both lumbar and cervical regions, regardless of the disease severity; the size of the lumbar spinal cord area directly correlates with the degree of urinary dysfunction, indicating structural reasons for clinical manifestations.

The study results show partial agreement with the findings of Vilchez et al., 3 who used DTI to show white matter damage in asymptomatic carriers. The study by Carvalho et al. demonstrates that straightforward MRI technologies can produce reliable and meaningful findings while maintaining high accessibility in areas with an endemic status for HTLV-1. The study demonstrates scalability because it operates with 1.5-T MRI technology combined with free Horos software in low-resource areas. The study includes blinded observers alongside inter-rater validation methods to boost both reliability and objectivity in the assessment process. The correlation between SC atrophy and the requirement for catheterization provides practical clinical value to medical practitioners.

The absence of automated tools or DTI analysis strengthens the study's translational value, according to the authors. The following areas, however, need future improvement: the study lacks longitudinal data, which prevents researchers from determining if probable HAM/TSP patients will develop into definite HAM/TSP cases; a respectable sample size of 101 participants is available, but a larger sample would be needed to validate the clinical decision threshold for lumbar SC area measurement at 0.417 cm ^2^ ; the absence of healthy control participants from the SC area threshold determination remains a notable gap. The research team includes seronegative subjects in their study, but normative MRI databases would have provided more robust interpretation results.

The paper presents evidence against the current assumption that MRI offers restricted usefulness in diagnosing HTLV-1 myelopathy during its early stages. 4 5 The implementation of simple, reproducible metrics by clinicians enables them to identify probable HAM/TSP patients who need closer medical attention and possible early interventions. The research advocates for MRI as a diagnostic tool beyond its traditional role of ruling out other conditions because it enables disease staging assessments. The findings help medical professionals connect patient symptoms to imaging test results, thus facilitating care in areas with limited resources. The paper presents evidence to modify diagnostic criteria for HAM/TSP by making objective MRI findings equal to or superior to the current distinction between probable and definite cases.

In summary, the study by Carvalho et al. provides valuable and applicable results that enhance the conventional MRI diagnostic abilities for HM/TSP. The paper extends previous DTI-based studies by enabling essential spinal cord imaging assessments in underserved areas. 6 7 8 Future research needs to establish the validity of lumbar spinal canal area thresholds while examining whether radiologic detection before therapy can enhance treatment results.

## References

[1] Yukitake M Takase Y Nanri Y Incidence and clinical significances of human T-cell lymphotropic virus type I-associated myelopathy with T2 hyperintensity on spinal magnetic resonance images Intern Med 2008 47 21 1881 1886 10.2169/internalmedicine.47.1284 18981631

[2] Dixon L McNamara C Dhasmana D Taylor G P Davies N Imaging spectrum of HTLV-1–related neurologic disease: a pooled series and review Neurol Clin Pract 2023 13 03 e200147 10.1212/CPJ.0000000000200147 37066106 PMC10092304

[3] Vilchez C Atrophy, focal spinal cord lesions and alterations of axial diffusivity in tropical spastic paraparesis J Neurovirol 2014 20 06 583 590 10.1007/s13365-014-0282-2 25227931

[4] Azodi S Nair G Enose-Akahata Y Imaging spinal cord atrophy in progressive myelopathies: HTLV-I-associated neurological disease (HAM/TSP) and multiple sclerosis (MS) Ann Neurol 2017 82 05 719 728 10.1002/ana.25072 29024167

[5] Bagnato F Butman J A Mora C A Conventional magnetic resonance imaging features in patients with tropical spastic paraparesis J Neurovirol 2005 11 06 525 534 10.1080/13550280500385039 16338746

[6] Romanelli L CF Miranda D M Carneiro-Proietti A BF Spinal cord hypometabolism associated with infection by human T-cell lymphotropic virus type 1(HTLV-1) PLoS Negl Trop Dis 2018 12 08 e0006720 10.1371/journal.pntd.0006720 30148843 PMC6128630

[7] Liu W Nair G Vuolo L In vivo imaging of spinal cord atrophy in neuroinflammatory diseases Ann Neurol 2014 76 03 370 378 10.1002/ana.24213 25042583 PMC4165784

[8] Morgan D J Caskey M F Abbehusen C Brain magnetic resonance imaging white matter lesions are frequent in HTLV-I carriers and do not discriminate from HAM/TSP AIDS Res Hum Retroviruses 2007 23 12 1499 1504 10.1089/aid.2007.0077 18160007 PMC2593463

